# Patient-level cost of home- and facility-based child pneumonia treatment in Suba Sub County, Kenya

**DOI:** 10.1371/journal.pone.0225194

**Published:** 2019-11-19

**Authors:** Joel Amenya Machuki, Dickens S. Omondi Aduda, Abong’o B. Omondi, Maricianah Atieno Onono

**Affiliations:** 1 Department of Research, Kenya Medical Research Institute, Kisumu, Kenya; 2 Department of Public Health and Community Development, University of Kabianga, Kericho, Kenya; 3 Department of Biomedical Sciences and Technology, The National University of Lesotho, Maseru, Lesotho; 4 Department of Biology, National University of Lesotho, Lesotho, South Africa; University of Ghana College of Health Sciences, GHANA

## Abstract

**Background:**

Globally, pneumonia accounted for 16% of deaths among children under 5 years of age and was one of the major causes of death overall in 2018. Kenya is ranked among the top 15 countries with regard to pneumonia prevalence and contributed approximately 74% of the world's annual pneumonia cases in 2018. Unfortunately, less than 50% of children with pneumonia receive appropriate antibiotics for treatment. Homa-Bay County implemented pneumonia community case management utilizing community health workers, as recommended by the World Health Organization (WHO), in 2014. However, since implementation of the program, the relative patient-level cost of home-based and facility-based treatment of pneumonia, as well as the main drivers of these costs in Suba Subcounty, remain uncertain. Therefore, the main objective of this study was to compare the patient-level costs of home based treatment of pneumonia by a community health worker with those of health facility-based treatment.

**Methods and findings:**

Using a cross-sectional study design, a structured questionnaire was used to collect quantitative data from 208 caregivers on the direct costs (consultation, medicine, transportation) and indirect costs (opportunity cost) of pneumonia treatment. The average household cost for the community managed patients was KSH 122.65 ($1.29) compared with KSh 447.46 ($4.71), a 4-fold difference, for those treated at the health facility. The largest cost drivers for home treatment and health facility treatment were opportunity costs (KSH 88.25 ($ 0.93)) and medicine costs (KSH 126.16 ($ 1.33)), respectively.

**Conclusion:**

This study demonstrates that the costs incurred for home-based pneumonia management are considerably lower compared to those incurred for facility-based management. Opportunity costs (caregiver time and forgone wages) and the cost of medication were the key cost-drivers in the management of pneumonia at the health facility and at home, respectively. These findings emphasize the need to strengthen and scale community case management to overcome barriers and delays in accessing the correct treatment for pneumonia for sick children under 5 years of age.

## Introduction

Globally, childhood pneumonia kills an estimated 880000 children yearly, accounting for 16% of all childhood mortality worldwide[1]. Half of the world's deaths due to pneumonia in children under the age of five years occur in Africa[2]. Kenya is currently ranked among the 15 countries with the highest estimated number of deaths due to childhood clinical pneumonia, with a mortality rate of 50.3 per 10,000 children under the age of 5 per year. Childhood pneumonia, when detected early, is easily amenable to basic prevention and control measures, most of which can be administered at primary care levels. Pneumonia case management is a high-impact treatment intervention delivered by various cadres of community health providers and is an important component of the Integrated Management of Childhood Illness (IMCI) strategy. It entails identifying and classifying pneumonia severity (as mild or severe) using clinical signs such as fast breathing, chest indrawing and general danger signs to guide the management approach[3]. Treatment includes home care advice, antibiotics for home therapy, or referral to a higher-level health facility. Mild pneumonia, characterized by fast breathing and/or chest indrawing, can be managed at home using oral amoxicillin, oral rehydration and bedrest, while severe pneumonia is characterized by any general danger sign that requires referral to a higher-level facility for appropriate parenteral and other supportive therapy [3]. Expanding treatment of mild pneumonia to the community level may not only improve access and compliance but also significantly reduce the economic burden on poor families. Evidence now shows that community case management (CCM) of suspected pneumonia with oral antibiotics can reduce pneumonia-specific mortality by 35% [4].

In 2006, Kenya adopted and launched the integrated community case management (iCCM) program as an integral component of the Integrated Management of Childhood Illness (IMCI) to accelerate the control and prevention of pneumonia, childhood diarrhea, malaria, neonatal mortality and malnutrition at the community level and to augment health facility-based case management. Pneumonia case management was first implemented in Kenya in 2014, with Homa-Bay County being the pilot site. The community health workers (CHWs) and the health care providers were trained on treating mild pneumonia in their respective community units based on the standard WHO guidelines for treating mild pneumonia. The CHWs are persons selected from the communities in which they live and work; they are selected by and answerable to the communities and are supported by the health system. The primary role of the CHWs is to serve as the households’ first point of contact with the health system. In Kenya, the CHWs work on a voluntary basis. However, the national community health strategy allows for them to be paid a stipend of up to KSH2500, which some county governments have now begun to pay to retain the CHWs within the health care system. The CHWs were assigned approximately 100 households in a community[5]. The CHWs were supplied with pneumonia treatment drugs to be dispensed at no cost to caregivers of children diagnosed with pneumonia for therapy at home[3,6,7]. Recent evidence has shown that some of the children with mild pneumonia still go directly to the health facilities, bypassing community health workers[8]. This is fraught with potentially high financial out-of-pocket costs to the patients and an increased risk of overcrowding at the health facilities. The preference for point of care and the potential role of attendant costs vary greatly with the prevailing context dynamics.

The estimated costs associated with the treatment of a pneumonia episode in children under the age of 5 can be divided into direct and indirect out-of-pocket expenses incurred by the household for an episode of pneumonia[9]. Direct medical costs are the actual amount paid by the households for consultation; they include investigations such as radiology, hospital admissions and medicines. Direct nonmedical costs are incurred for transportation and meals for caregivers at the health facility. Indirect costs are the opportunity cost of caregiver time and foregone wages during the time of seeking care for the child[9,10]. Information on whether pneumonia point-of-care cost differences are a key determinant of the child caregiver’s choice of preferred place to seek help is still mostly scarce in most counties in Kenya. Most studies the estimating household cost of pneumonia care have focused on costs that child caregivers incur while seeking services at health facilities, and others have focused on the cost incurred by the ministry of health in managing pneumonia. There is limited documentation indicating the cost the caregivers may incur while seeking care at the community level from CHWs. Therefore, there was a need to conduct this study that focused on estimating and comparing the cost incurred by the caregivers when seeking care either in the community from the CHWs or at the health facility among children aged 2–59 months in Suba Subcounty in western Kenya.

## Methods

### Setting

This study was conducted in Suba Subcounty, Homa-Bay County in Kenya, where pneumonia community case management was piloted in 2014 and 2015. Homa-Bay County is located in the Nyanza region near Lake Victoria. Suba Subcounty was a rural setting with a population of approximately 119,000 people, of whom 17900 were children under the age of 5. Suba Subcounty had a child mortality of 130 deaths per 1000 live births[11]. It had 18 health facilities and 240 integrated community case management (iCCM) trained CHWs working in the 26 community health units (CHUs). Each CHW was in charge of 500 people (between 50 and 100 households). The CHW submitted monthly reports to the community extension workers that were transmitted to the County health information systems.

### Study design

A descriptive cross-sectional design utilizing quantitative methods was performed to assess the cost of treatment of mild pneumonia at the health facility versus at home based on a national community pneumonia case management protocol[3]. The study was nested within an implementation study for integrated community case management of pneumonia that was conducted in Homa-Bay County Suba Subcounty and whose detailed methodology has been published[6,7].

### Participants

The study sample was identified from selected community health units in Suba Subcounty that participated in the parent iCCM study. All 26 community units in Suba Subcounty were selected to participate in the study. Participants were consenting caregivers (including mothers, fathers, grandmother and guardians) of children aged 2–59 months whose children had been treated for pneumonia in the last month; the participants all sought care for their children at the health facility (service providers are often either clinical officers or nursing officers) or from a CHW at home.

### Study sample size

The number of children who developed pneumonia during the study period was assumed to be 358, since the incidence of pneumonia among children under 5 years old in Suba was 2% and therefore, out of 17900 children under years old registered in the iCCM midline survey in Suba Subcounty, it was expected that 358 new cases of pneumonia developed. Therefore, the estimated sample size, with a 5% margin of error and a 95% confidence level, was 189. With the addition of 10% loss, the final total sample size became 208 participants.

The participants were selected from each of the community health units (CHUs), ensuring that 50% of participants will be caregivers who sought care at the health facility and the other 50% sought care at home from a CHW.

### Data sources

Data were collected using a validated questionnaire similar to one used in a study in Pakistan[12]. Data for the cost pneumonia treatment were collected from a household perspective. Data on household demographics, income, and out-of-pocket expenses incurred by the caregiver during the treatment of the pneumonia episode were collected from caregiver interviews. The caregivers who sought care at the health facility were traced using their phone numbers retrieved from the registry of patients under the age of 5 at the outpatient departments. Caregivers were called and visited in their homes where the interviews took place. The caregivers who sought care from a CHW were interviewed on the day 14 follow-up visits by a research assistant who traced them using the caregiver’s phone number retrieved from the sick child recording form.

#### Concepts to be measured in pneumonia treatment cost

The patient-level cost of treatment was divided into two major categories: direct and indirect costs. The direct costs were further divided into direct medical costs and direct nonmedical costs. Direct medical costs included the amount of money paid by the households or caregiver for consultations, laboratory tests and radiology. This cost was derived from the payment receipts for these services. The cost of drugs prescribed was valued by the retail price. Direct nonmedical costs included costs incurred for transportation, meals for caregivers at the health facility, and payments made for administrative services at the health facility, which included costs such as buying prescription booklets for the clinicians or paying registration fees[13].

Indirect costs included the opportunity cost of caregiver time and foregone wages measured at the household level. Opportunity cost was estimated as the approximate value of nonwage household activity to account for time spent on care seeking and childcare based on the assumed expected earnings if the person was working. For calculating time cost, lost minutes were recorded and converted into numbers of working days. The mean monthly income of the head of the household was converted into daily income, and the opportunity cost was calculated as days lost multiplied by the mean daily income of the head of the household. Foregone earnings due to absence from work to take a child for treatment were self-reported by the households[14].

### Statistical methods

The data were analyzed using IBM Corp. Released 2011. IBM SPSS Statistics for Windows, Version 20.0. Armonk, NY: IBM Corp.. A descriptive analysis was performed for the costs and the subsequent health seeking behaviors, and the results are presented as frequencies, means, standard deviation (SD) and median. The bivariate analyses comparing the total treatment costs covered by households when seeking pneumonia treatment from community health workers at home versus treatment at the health facility was performed using *t*-test procedures. The *p*-values for both direct cost and indirect costs of treatment were reported.

### Ethical considerations

Ethical approval for this study was obtained from the Kenya Medical Research Ethics Review Committee. In carrying out the study, written **i**nformed consent was sought from the participants with full information being provided and comprehension being affirmed. Confidentiality was ensured through anonymity (using unique numbers) and privacy during interviews; study withdrawal at any point was permitted.

## Results

### Sample characteristics

A total of 204 (104 treated by the CHW and 100 treated by medically trained health facility workers who were usually clinical officers or nursing officers) caregivers of children with pneumonia were enrolled in the study. The mean age of the children whose caregivers were interviewed was 2 years (SD 1.45). The majority of the caregivers interviewed in both groups were mothers; most of the mothers had only primary-level education while the fathers, on average, had obtained either secondary or postsecondary education. The household income was higher for families who sought pneumonia treatment at health facilities than those who sought care from CHWs. Less than 40% of the overall participants were formally employed. In both groups, a larger proportion of fathers than mothers had a secondary school-level education or more (mothers 31% vs. fathers 99% and mothers 28% vs. fathers 54% for the households that sought care at the health facility and CHW, respectively). The median household monthly income was KSH 9000 ($94.7) IQR (KSH8000-20000) ($84.2-$210.5) and KSH 19600 ($ 206.3) IQR (KSH6500-45000) ($68.42–473.6) in the groups treated by CHW and health facility workers, respectively (Table 1).

**Table 1:** Sociodemographic characteristics of children seeking pneumonia treatment from CHWs and health facilities in Suba Subcounty, Kenya.

Participant Characteristics			Facility-based Management N = 100	Home-based management N = 104	*χ*^*2*^ *value*	P-value
Respondent relationship to the child		Father Mother	9 (9)	0	0.1645	0.921
			91 (91)	0		
Education level (Mother’s)	None		6 (6)	7 (7)	2.6	0.76
	Primary		63 (63)	68 (65)		
	Secondary		26 (26)	25 (24)		
	Postsecondary		5 (5)	4 (4)		
Education level (Father’s)	Primary		1 (1)	49 (46)		
	Secondary		42 (42)	46 (44)		
	Postsecondary		57 (57)	9 (10)		
	Mother Yes		7 (7)	10 (10)	1.6	0.203
	No		93 (93)	94 (90)		
Parents formally employed	Father Yes		37 (37)	40 (38)		
	No		63 (63)	64 (62)		
Household monthly income, median (IQR)			19600 (6500–45000)	9000 (8000–20000)		

Note: please note that the figures in brackets are a percentage of N

### Aspects of pneumonia treatment cost

#### a. Direct nonmedical cost

The mean nonmedical cost for the clients who sought care from CHWs was Kenya shilling (KSH) 22.9 ($0.2) (SD 19.57 ($0.2) and range KSH 10–67 ($0.1–0.7)) compared to those who sought care at health facility who had a mean cost of KSH 43.0 ($0.45) (SD 29.21 (0$0.3)). There was no statistically significant difference in transportation cost between clients who sought care at health facility and those who sought care from CHWs (P>0.08, t value and df = 202), as shown in the table below (Table 2).

**Table 2:** Comparison of the direct nonmedical costs of pneumonia treatment among caregivers who sought care from CHWs and health facility in Suba Subcounty.

Cost Component	Health Facility (HF)	CHW	95% CI of difference	t-value (df)	P-value
	
Transportation Costs in KSH	43.0 (29.21)	22.86 (19.57)	2.91–44.04	2.56 (202)	0.08

Number of cases: HF-health facility = 100; CHW-community health worker = 104; degrees of freedom = 204

Note: Figures in brackets are standard deviations.

#### Indirect costs

The indirect costs were estimated as the opportunity costs of caregiver time. The average time spent by caregivers was 1.2 hours for the CHW group and 1.8 hours for the health facility group. The average opportunity cost incurred for caregivers who sought care at the health facility was KSH 155.2 ($1.6), while the cost incurred for care from the CHWs was KSH 88.3 ($0.9).

### Cost of pneumonia treatment by the CHWs versus the health facility

The total cost of treatment of pneumonia when seeking care from the CHWs was KSH 122.65 ($1.29). The largest cost drivers were the indirect costs, which contributed KSH 88.25 ($0.93) (comprising the opportunity cost of caregiver time), followed by the direct nonmedical cost of KSH 22.86 ($0.24) (including transportation), and the direct medical cost, KSH 11.54 ($0.12), which was the cost of medicine. Among the group that sought care from the CHWs, 100% of the direct medical costs were incurred from the medicine. The consultation was free so no cost was incurred. The average cost of treatment of pneumonia when seeking care at the health facility was KSH 447.46 ($4.7). The largest cost drivers were the direct medical costs, which contributed an average of KSH 248.89 ($2.62) (comprising medicine, laboratory tests and consultation); the additional average indirect cost component was KSH 155.15 ($1.63) (consisting of the opportunity costs). The lowest cost driver when seeking care at the health facility was the transportation cost of KSH 43.42 ($0.46). The health facility costs were distributed as follows: consultation 12.05%; medicine 50.69% and laboratory test 37.26%. There was a statistically significant difference in the mean average cost for consultation and medicine (p< 0.0001) between the group that sought care from the CHWs and the group that sought care at the health facility. There was no comparison for the other costs, such as laboratory tests, radiology and admission, because these costs were incurred only in the group that sought care at the health facility (Table 3).

**Table 3:** Comparison of the cost drivers for direct medical costs among caregivers that sought care at CHW and health facility in Suba Subcounty.

Cost Component	Whole Sample Mean	HF Mean	CHW Mean	95% CI for difference	P-value
Consultation	15.55 (23.30)	30.0 (26.10)	0 (0.00)	23.81–33.98	<0.001
Medicine	47.72 (91.25)	126.16 (105.50)	11.54 (29.96)	52.94–179.33	<0.001
Lab test	-	92.73 (57.62)	-	-	-

Number of cases by facility: HF-health facility = 100; CHW-community health worker = 104; degrees of freedom = 204

Figures in brackets are standard deviations.

## Discussion

The overall aim of this study was to compare the patient-level costs of home-based CHW treatment of pneumonia versus health facility-based management among children aged 2–59 months who were diagnosed with pneumonia in Suba Subcounty, western Kenya. The study only focused on those children who were treated for simple pneumonia based on the WHO guidelines, where both sources of care offered a standard prescribed outpatient treatment. To the best of our knowledge, this is the first study conducted in Kenya to estimate the cost of pneumonia treatment at the community level from the household perspective.

The study found that there was a substantially lower household average income for caregivers who sought treatment at home from the CHWs compared to those who sought care at the health facility. The average household cost for the community healthcare worker managed cases was KSH 122.65 ($1.29) compared with KSH 447.46 ($4.71) for those treated at the health facility. This difference is quite important for poor areas with limited resources, such as Homa-Bay County, which records a high poverty index; 48% of the population live below the poverty line. The cost difference was caused by both direct (consultation, medicines, bed charges, and transportation) and indirect (opportunity cost and lost earnings) costs. The pattern also reflects care-seeking behaviors based on household income inequities, which is inconsistent with the national referral strategy and iCCM management concepts.

This study found that direct medical home-based treatment costs for pneumonia were KSH 0 ($0.00), which is similar to a study by Matovu *et al*. (2014) in Uganda who also found zero household direct medical costs of treatment of pneumonia for the caregivers who sought care at home[15]. The medicines that were offered at the community level were issued free of charge through donor contribution toward implementation of integrated community case management.

The direct nonmedical costs incurred by those who sought care in the health facilities in our study primarily involved the incurred cost of transportation to and from the point of care. Comparing our study with that of Matovu *et al*. (2014), there was some difference in the total time lost by households in the group that sought care from the CHWs (72.6 minutes; the opportunity cost was KSH 88.25 ($0.93) in relation to Matovu and others who found that the average time lost when seeking care from the CHWs was 27 minutes[15]. This can be attributed to a difference in access to the CHWs. The study settings seemed to have a difference in topographical and infrastructural differences.

According to Ayieko’s study on the economic burden of pediatric care, which was conducted in National and provincial hospitals in Kenya, the mean total cost of treatment of pneumonia was KSh $12.54 compared to ($4.71) in our study[14]. This was significantly higher compared to our study, indicating an approximately 3-fold difference in cost. Although both studies were conducted in Kenya, the health facilities utilized were of different levels. Ayieko utilized National and provincial (current county) level hospitals, while our study utilized dispensaries and health centers (level 1 and level 2 health facilities), where direct medical costs are inexpensive by policy design. The largest cost driver for the treatment of pneumonia in Ayieko’s study was consultation costs, unlike our study, where medicine was the highest cost driver. In comparison with other studies[16,17], the largest cost driver for treatment of pneumonia at the health facility was medicine ($22.64). These costs were 12-fold greater than the cost incurred in this current study. Differences in the cost of medicine can be linked to a subsidized treatment cost for children under the age of 5 in government facilities in Kenya[18]

The inherent limitation of this study was its cross-sectional nature. It is probable that the selected women may not be a good representation of the population, which limits the generalizability of our findings. Another limitation was that we only used quantitative methods in this evaluation. While quantitative methods are effective in measuring associations, they are less effective in providing a deep understanding of the processes and how those changes actually occur. A mixed methods approach allowing triangulation of qualitative and quantitative findings would have provided a deeper understanding of the drivers of health seeking preferences, satisfaction and perceived quality of the services. While we did not conduct qualitative work, a substudy within the parent project compared the perceived quality of services and health seeking behaviors among those seeking care from CHWs versus health facilities[19]. This substudy found that caregivers perceived CHWs to provide higher quality care in terms of accessibility and patient relationship and equal quality care in terms of clinical aspects[19]. Our results are therefore complimentary to this previous study and support that notion that iCCM for pneumonia is cost-effective and acceptable.

## Conclusion and recommendations

Pneumonia is one of the major causes of death among children < 5 years of age in developing countries, including Kenya. This study demonstrates that the costs incurred for community-managed pneumonia treatment are considerably lower compared to facility-based pneumonia management. This finding has a potential profound impact on infant morbidity and mortality. Extending severe pneumonia treatment to the community level, will not only improve access and treatment outcomes but will also decrease the economic burden on the families. Extending services to the community will also decrease pressure and cost on the already overburdened public health system. Ultimately, integrated community case management of pneumonia can accelerate Kenya’s progress toward achieving SDG 3 and achieving universal health care for children under 5 years of age. Therefore, this study recommends strengthening simple pneumonia treatment at the community level as a cost-effective measure to improve access to treatment. We further recommend that the county and national governments explore ways to minimize travel and opportunity costs, which were the key cost drivers for patients/caregivers, to reduce the pneumonia cost burden.

Second, the number of patients bypassing the primary care level to attend health facilities for the treatment of simple pneumonia is still considerable. This finding is positive in that community case management is complementary to health facility management. Therefore, this finding allays the fears of those who may think that community case management competes and diverts patients from health facilities. We recommend further research and approaches to improving linkages between the CHWs and health facilities. However, because it is plausible that caregivers were not aware that CHWs can diagnose and treat pneumonia at home, we recommend community sensitization on the utilization of pneumonia community case management services, which reduces the household out of pocket expenditure. Further, there is a need to conduct further studies to measure the quality of care both at the health facility and at home with community health workers.
